# Economic evaluation of preventive cognitive therapy versus care as usual in cognitive behavioral therapy responders

**DOI:** 10.3389/fpsyt.2023.1134071

**Published:** 2024-01-10

**Authors:** Margo de Jonge, Matthijs Blankers, Claudi L. H. Bockting, Maarten K. van Dijk, Martijn J. Kikkert, Jack J. M. Dekker

**Affiliations:** ^1^Department of Research, Arkin, Amsterdam, Netherlands; ^2^Novarum, Amstelveen, Netherlands; ^3^Trimbos, Utrecht, Netherlands; ^4^Amsterdam University Medical Centre, Amsterdam, Netherlands; ^5^Dimence Mental Health Care Centre, Deventer, Netherlands; ^6^Department of Clinical Psychology, Vrije Universiteit, Amsterdam, Netherlands

**Keywords:** economic evaluation, cost-effectiveness, randomized controlled trial, relapse, recurrence, depression, cognitive therapy

## Abstract

**Background:**

The highly recurrent nature of Major Depressive Disorder is a major contributor to disability and health care costs. Several studies indicate that recurrence may be prevented with Preventive Cognitive Therapy (PCT). This study is the first to perform an economic evaluation of PCT in comparison with care as usual for recurrently depressed patients who experienced two or more depressive episodes and remitted after receiving Cognitive Behavioural Therapy.

**Methods:**

An economic evaluation from the societal perspective was performed alongside a randomized trial (*N* = 214). Health-related quality of life (QALYs), depression-free days, health care utilization, and productivity losses were measured between randomization and 15 months follow-up. The costs were indexed to the reference year 2014.

**Results:**

QALY gains did not differ significantly between the groups (*p* = 0.69). Depression-free days were higher after PCT (*p* = 0.02). Societal costs of PCT were 10,417 euro and for care as usual 9,545 euro per person. We found a 47% likelihood that PCT led to additional QALYs at higher costs, and there was a 26% likelihood that PCT led to fewer QALYs at higher costs. When depression-free days was used as an outcome, we found PCT had a 72% likelihood of leading to more depression-free days at higher costs than care as usual and a 27% likelihood of leading to more depression-free days at lower societal costs.

**Limitations:**

The 15-month follow-up might be too short to draw long-term conclusions about the cost-effectiveness of the PCT. The data collected for this study is part of an RCT to examine the effectiveness of adding PCT to care as usual. Therefore, the study was powered primarily to detect an effect in time to relapse/recurrences.

**Conclusion:**

The economic evaluation is slightly in favour of the PCT condition when depression-free days is used as an outcome. PCT is not cost-effective given the high costs per additional QALYs from the societal perspective when QALYs are the effect measure.

**Clinical trial registration:**

https://www.onderzoekmetmensen.nl/en, identifier NL2482.

## 1 Introduction

Major Depressive Disorder (MDD) has a significant impact on individuals and negatively affects many aspects of life (1). The annual prevalence in the general population varies from 4% to 6% and epidemiological estimates place the lifetime prevalence of MDD at more than 16% (2–4). The major contribution of MDD to disability and health care costs is largely due to its highly recurrent nature (5, 6). But next to the depressive episodes, residual symptoms after remission and loss of productivity are also important factors contributing to the considerable health care costs (7–9). Due to limited resources in many health care systems, cost of treatment is of great concern. To overcome these financial constraints, it is important to develop accessible, acceptable, time-efficient, and economically affordable treatments for patients suffering from recurrent depression. Therefore, cost-effective preventive interventions are beneficial from the viewpoint of patients and society at large.

The majority of individuals with MDD experience more than one episode and the risk of another episode increases with each relapse or recurrence (10). For high-risk groups, reported relapse and recurrence rates rise to 60%–70% over a 2-year period (11, 12). Therefore, improvement of long-term outcomes is important in the treatment of MDD. A form of cognitive therapy aimed specifically at preventing relapse and recurrence is Preventive Cognitive Therapy (PCT) (11). PCT has been proven effective in preventing relapse and recurrence in patients with multiple episodes over 5.5–10 years compared to treatment as usual (11, 13). In a three-arm RCT comparing maintenance antidepressants to tapering antidepressants with PCT versus the combination of maintenance antidepressant drugs (AD) and PCT, maintenance AD was not superior to PCT administered while tapering off AD (relapse/recurrence risks over 15–24 months: 60% versus 63%) (14). Adding PCT to maintenance AD reduced relapse risk with 41% as compared to maintenance AD alone (14).

Up till now, only a few studies have focused on the cost-effectiveness of PCT aimed at preventing relapse and recurrence of remitted patients. First, supported self-help of PCT after remission was found to be effective but not cost-effective compared to treatment as usual (15). They examined both health-related quality of life (QALYs) and relapse/recurrence as outcomes. For relapse/recurrence, at a willingness to pay (WTP) of 22,000 euro per recurrence prevented, the probability that self-help PCT being cost-effective, in comparison to treatment as usual, was 80%. For QALYs, at a WTP of 30,000 euro per QALY gained, the probability of PCT being cost-effective is 21% (15). Second, an internet-based form of PCT was neither effective nor cost-effective compared to treatment as usual alone (16). They examined both QALYs and depression-free days as outcomes and found no differences between internet-based PCT and treatment as usual (16). However, the most recent study performing an economic evaluation of AD versus PCT with or without tapering of AD concluded that adding PCT to AD was cost-effective compared to AD alone (17). They examined both QALYs and depression-free days as outcomes. Adding PCT to AD was cost-effective compared with AD only regarding depression-free days and resulted in lower costs at the population level. They found a 93.1% probability that costs were lower and health outcomes better for PCT combined with AD compared with AD alone (17).

Until recently, it was unknown whether the addition of PCT to care as usual would be more effective than care as usual alone for patients who remitted after Cognitive Behavioral Therapy (18) Since PCT is based on Cognitive Behavioral Therapy, it is possible that PCT might not provide any additional prophylactic effect in patients that already received Cognitive Behavioral Therapy during the acute phase of treatment. In this study, it was found that offering subsequent PCT to patients who responded to acute phase Cognitive Behavioral Therapy was effective compared to care as usual in delaying the time to relapse/recurrence of depression over a period of 15 months (19). However, the cost-effectiveness of PCT after responding to acute Cognitive Behavioral Therapy is still unknown. The aim of the current study was to perform an economic evaluation alongside the RCT (19), in which the addition of PCT to care as usual was compared to care as usual alone in delaying the time to relapse/recurrence over a period of 15 months in patients who remitted after Cognitive Behavioral Therapy.

## 2 Methods

### 2.1 Design

A detailed description of the design of the RCT is available elsewhere (18) but is briefly summarized below. Methods for the economic evaluation are based on the Blue book by Drummond et al. (20) and follow the International Society For Pharmacoeconomic and Outcomes Research (ISPOR) guidelines on economic evaluation alongside RCTs (21). Reporting of the economic evaluation follows the Consolidated Health Economic Evaluation Reporting Standards (CHEERS) statement (22). Medical ethics approval for the RCT has been obtained (METiGG: NL34721.097.10) and the study was conducted in compliance with the Declaration of Helsinki (23).

### 2.2 Participants

The target population consisted of patients with a history of two or more major depressive episodes (MDEs), which is a group with an elevated risk for relapse and recurrence of depression. Patients were recruited between January 2012 and August 2014 in the Netherlands. All received written information about the study and were asked to sign the informed consent.

Patients were included if they (a) had at least two previous MDEs, (b) were currently in remission according to DSM-IV criteria for at least two months as assessed by the Structured Clinical Interview for DSM-IV Axis I Disorders (SCID-I) (24), (c) had no-to-mild depressive symptoms defined as a current score of <14 on the 17 item Hamilton Depression Rating Scale (25), (d) had received prior cognitive therapy, with a minimum of eight sessions, (e) are fluent in Dutch, (f) had no current or history of bipolar illness or any psychotic disorder, (g) had no current alcohol or drug misuse, (h) had no acute predominant anxiety disorder, and (i) provided informed consent.

### 2.3 Treatment

#### 2.3.1 Preventive cognitive therapy

After remission of depression, there was a minimum two-month waiting period before Preventive Cognitive Therapy (PCT) started. PCT consisted of eight individual sessions once a week, offered as a sequential treatment after response to acute Cognitive Behavioral Therapy. PCT is an adapted type of Cognitive Therapy specifically developed to prevent relapse in recurrent depression (11). Each PCT session follows a fixed structure, with agenda setting, review of homework, explanation of the rationale of each session, and assignment of homework. Unlike Cognitive Therapy, PCT is not primarily directed toward modifying negative thoughts. Instead, the main focus is on dysfunctional beliefs (attitudes and schema) using specific challenging techniques with the help of positive phantasy. Specific attention is paid to enhancing the memory and retrieval of positive experiences and making a personal prevention plan. A specific manual for the client and therapist has been published describing the structure of the treatment (11).

The PCT therapists were trained psychologists who specialized in Cognitive Behavioral Therapy, all completed the basic Cognitive Behavioral Therapy course which has a minimum of 1-year training. Furthermore, they received a two-day training in PCT from the author of the PCT protocol. During the study, the therapists participated in regular supervision groups led by the trainer, which was held once a week. The therapists who performed the PCT are not the same therapists that provided the acute phase Cognitive Behavioral Therapy.

#### 2.3.2 Care as usual

Care as Usual (CAU) consisted of usual care that patients receive in primary care and in secondary care after acute Cognitive Behavioral Therapy for depression. CAU could be no treatment at all or anti-depressant maintenance medication, which was in most cases was provided by a general practitioner.

### 2.4 Measurements

Every participant was followed-up over a period of 15-months. The follow-up assessments were self-report questionnaires at 3-, 6-, 12- and 15-months post-randomization. The assessments at baseline and at the 15-month follow-up consisted of an interview and self-report measures. All interviews were done by trained assessors who were blinded to the allocation of the participants and who attended regular consensus meetings.

### 2.5 Costs

For the economic evaluation, we took the societal perspective as our base case perspective. Therefore, all costs related to the PCT and CAU interventions, other health care uptake, and productivity losses or gains were included. All costs (Table 1) are expressed in euros and were indexed to the reference year 2014 using an inflation and price level correction based on the Harmonized Index of Consumer Prices (HICP) (26).

**Table 1:** Patient demographic and clinical characteristics at time of randomization.

Characteristic	CAU (*n* = 107)	PCT (*n* = 107)
Sociodemographic		
Age, mean (S.D.)	44.7 (11.3)	42.1 (11.2)
Gender, no (%)		
*Male*	37 (34.6)	31 (29.0)
*Female*	70 (65.4)	76 (71.0)
Marital status, no (%)		
*Single*	62 (57.9)	65 (60.7)
*Married or registered partnership*	35 (32.7)	31 (29.0)
*Divorced*	10 (9.3)	7 (6.5)
Education, No (%)		
*Low*	10 (9.3)	11 (10.3)
*Intermediate*	33 (30.8)	34 (31.8)
*High*	64 (59.8)	58 (54.2)
Clinical		
Patients on antidepressants, no (%)	34/100 (34)	23/100 (23)
Previous MDEs, median (25th, 75th percentile)	3 (2, 4)	3 (2, 5)
Age of first onset, mean (S.D.)	25.8 (12.8)	24.3 (10.6)
Severity last MDE, no (%)		
*Mild*	12 (11.2)	11 (10.4)
*Moderate*	49 (45.8)	56 (52.9)
*Severe*	46 (43.0)	39 (36.8)
HRSD-17 T0, mean (SD)	4.52 (3.89)	4.25 (3.54)
Days to relapse/recurrence	362.3 (152.4)	408.7 (103.3)

CAU, care as usual; PCT, preventive cognitive therapy; MDE, major depressive episode; Severity last MDE as assessed by Structured Clinical Interview for DSM-IV Axis I Disorders, Mild = 5 symptoms, Moderate = 6–7 symptoms, Severe = 8–9 symptoms, HRSD-17 = 17-item Hamilton Rating Scale for Depression, Time is presented in days.

Health care utilization and productivity losses were measured using the Trimbos Questionnaire for Costs associated with Psychiatric illness (TIC-P) (27). The TIC-P is a self-report measures that measures health care utilizations regarding mental health care, other health care and medication. It also measures productivity losses (absenteeism and presenteeism). Using the TIC-P, the number of contacts with health professionals and amount of informal care was collected for four weeks. This reported number of contacts was extrapolated to the period between the current and previous measurement wave.

To measure the cost of health care, we considered all health care received by the patient, including the costs of PCT/CAU. We used a societal perspective according to the Dutch guidelines for health economic research. Dutch standard cost prices were used to value resource utilization (28) by multiplying the number of contacts with the standard cost prices per contact. Medication costs were valued based on the reports of medication use by the trial participants, collected during the assessments. Frequency of use was assumed to be daily if these data were missing. Cost prices per dose of medication were extracted from the Netherlands Ministry of Health maximum cost prices of medication registry (29). Medication costs represent the combined costs for AD, other psychiatric medication, and medication for somatic illnesses.

Using the Short Form-Health and Labor Questionnaire (SF-HLQ), a subscale of the TIC-P, both absenteeism and presenteeism were measured. Together, these measures comprise our measure of productivity losses. Productivity losses were reported by the patients over the two weeks prior to the day of data collection, as per the TIC-P instructions. The reported hours/days of productivity losses over the two weeks were then extrapolated to the period between the current and previous measurement wave. Productivity losses in hours were multiplied by an estimate of labor costs of 37.90 euro for men and 31.60 euro for women, respectively (28). Productivity losses were valued using the friction cost method. A maximum friction costs period of 85 days and an elasticity factor of 0.8 were applied (30).

### 2.6 Effects

The outcome measure for the cost-utility analysis (primary analysis) was the number of quality-adjusted life years (QALY) gained or lost between randomization and the 15-month follow-up. The 5-dimensional, 3-level EuroQol (EQ-5D-3L) quality-of-life instrument was used. The EQ-5D-3L is a self-report instrument which was in this study used to calculate health utilities based on the Dutch tariff (31) were calculated by multiplying the utility of a health state by the time spent in this health state using linear interpolation between measurement time points.

The outcome measure for the cost-effectiveness analysis was the number of depression-free days until relapse/recurrence. The number of depression-free days was calculated by using the date of baseline measurement and the date of relapse/recurrence within the 15-month follow-up period. Relapse/recurrence was operationalized as meeting DSM-IV criteria for a MDE according to the Structured Clinical Interview for DSM-IV Axis I Disorders (SCID-I) over a follow-up period of 15 months (24). The SCID-I is considered to be the gold standard in semi-structured instruments for depression. For the Dutch version of the SCID-I the inter-rater reliability has been shown to be fair to excellent (32). The SCID-I was administered at baseline and at follow-up 15 months later.

### 2.7 Data preparations

We carried out all analyses following the intention-to-treat paradigm. Missing observations in costs and effects data were handled using multiple imputation in the base case scenario, meaning that all data in the tables are based on multiple imputed data unless otherwise indicated. The multiple imputation software package we used is Amelia II (33) for R (34). We have used this package as there are indications that Amelia II handles non-gaussian distributions relatively well (35). Using this software, we imputed the original dataset 10 times, which is sufficient given the low overall rate of missing data in the dataset used for the economic evaluation (6.5%, range 0%–13%) (36). Cost parameters were square root transformed before imputation and back-transformed afterwards to take in account the skewed distribution of these variables because Amelia assumes multivariate normal distribution of data. Analyses were performed on each of these 10 imputed datasets separately, and the outcomes were then combined using Rubin’s rules for combining means and standard errors from multiple imputed datasets (37, 38).

### 2.8 Cost-effectiveness calculations

For all participants, we multiplied units of health care (e.g., sessions, contacts, and medication), time investments, and productivity losses by their associated costs. Differences in costs and effects between PCT and CAU were calculated as the difference in cumulative costs over the 15-month time horizon of this study. There was no need to apply baseline correction as randomization had resulted in sufficient comparability across conditions at baseline. All costs were standardized using Organization for Economic Co-operation and Development purchasing power parities for base year 2014. As the time horizon of the study was only a little over a year, no future costs / effects discounting has been applied. As for the costs perspective, the societal perspective was chosen in the base case scenario, while the health care sector perspective is presented the alternative scenario. In order to assess the sensitivity of our findings to misspecification of costs, one-way sensitivity analyses were performed to evaluate the impact on the ICERs of a − 20% to +20% misspecification in the cost categories.

**Figure 1:**
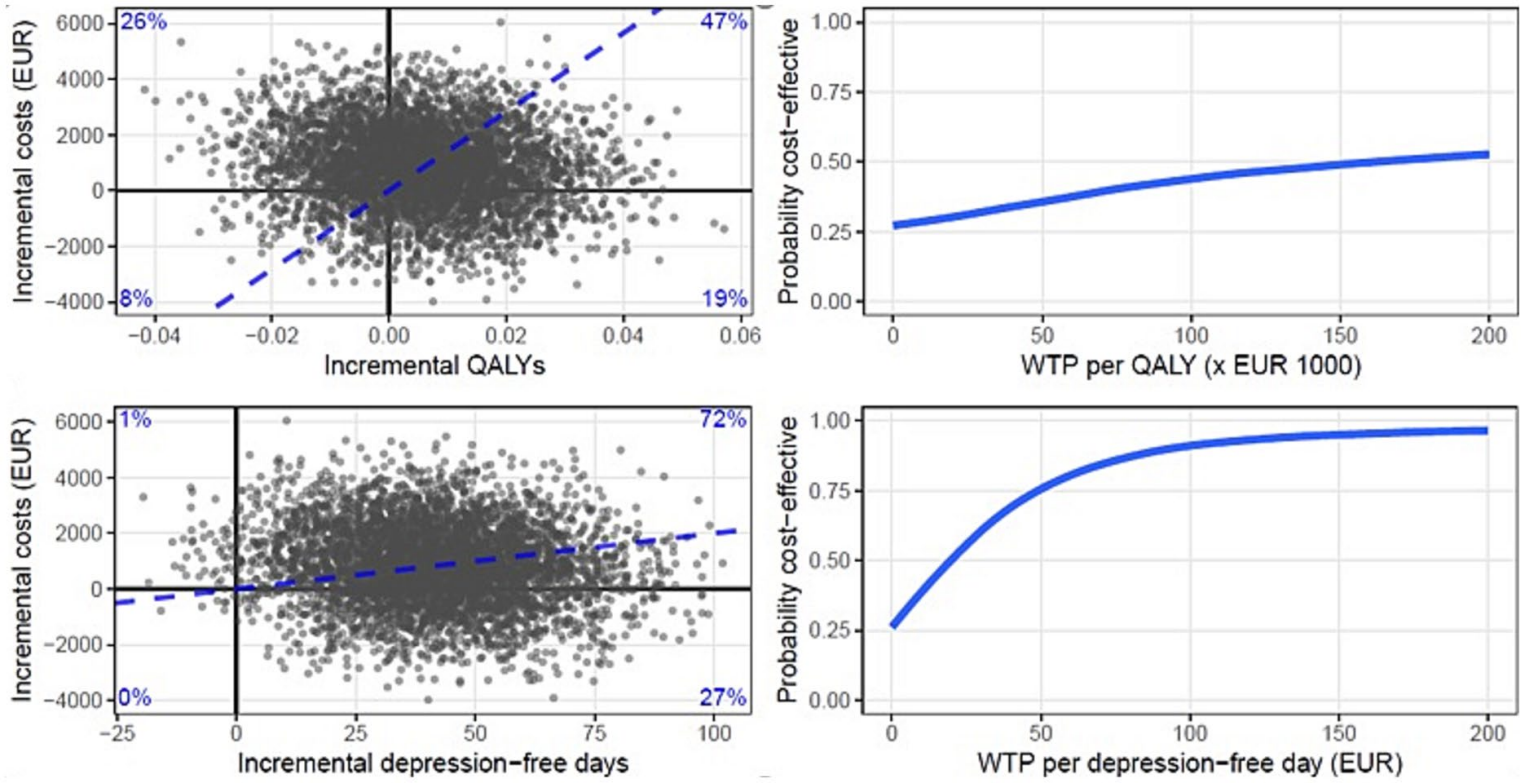
Cost-effectiveness planes and cost-effectiveness acceptability curves (CEAC) of the base case scenario with QALYs (top) and depression-free days (bottom) as the effect parameters. Each dot in the plane represents a bootstrapped mean ICER. The CEAC show the probability of PCT being more cost-effective as a function of the willingness to pay in Euros.

We extracted 5,000 nonparametric bootstrapped samples (with several patients per trial arm equal to the number of patients in the original dataset) from the MI dataset (500 per imputed data set). For each of these 5,000 bootstrapped samples of the dataset, we calculated the incremental costs, incremental effects, and incremental cost effectiveness ratio (ICER). The ICER was calculated as: ICER = (CostsPCT – CostsCAU) / (EffectsPCT – EffectsCAU), where effects were either QALYs or depression-free days. The ICERs were used for further calculations and plotted on the cost-effectiveness plane. The reference intervention (CAU) is positioned in the origin of the cost-effectiveness plane. The horizontal axis indicates differences in health gains between PCT and CAU, while the vertical axis represents differences in costs. Along the horizontal and vertical axis, Figure 1 is divided into quadrants, each with a specific interpretation. ICERs that fall in the upper right (“North East”) quadrant indicate that PCT generated better health at additional costs; the lower left (“South West”) quadrant indicates fewer health gains from PCT than CAU at lower costs. In the upper left (“North West”) quadrant, PCT is dominated by CAU, as poorer health outcomes are then obtained at additional costs. In the lower right (“South East”) quadrant, PCT dominates CAU with better health outcomes against lower costs.

Based on the distribution of the ICERs over the cost-effectiveness plane, cost-effectiveness acceptability curves (CEACs) (39) have been drawn (Figure 1). CEACs show the probability that PCT is more cost effective than CAU as a function of the willingness to pay (WTP) for 1 additional unit of effect (1 QALY or 1 depression-free day). At a probability of 0.5 on the vertical axis, the indifference point is reached. Above this indifference point, PCT has a better likelihood to be preferred over CAU with regard to cost-effectiveness (with a likelihood equal to the probability on the vertical axis). As the WTP per unit of effect is generally an unknown quantity it is presented as a series of increments on the horizontal axis.

## 3 Results

### 3.1 Descriptive analysis

A total of 214 patients were included in the RCT. The majority (68%) were women, and the mean age was 43.4 years. The mean number of previous depressive episodes was 3.98 and 28.5% of patients used antidepressant medication (AD) at baseline. None of the baseline characteristics differed between the Preventive Cognitive Therapy (PCT) and care as usual (CAU) conditions, see Table 1.

### 3.2 Costs

Table 2 presents the costs for the different cost categories. There was no difference in total societal costs during the 15-month follow-up period, with a bootstrapped mean difference of 858 euro (95% CI −5 to 1753).

**Table 2:** Cost per cost category and differences in costs between CAU group and PCT group (in euros) over 15 months.

	CAU group (*n* = 107)	PCT group (*n* = 107)	CAU group vs. PCT group
	Mean euro (sd)	Mean euro (sd)	Mean difference euro (95% CI)
Health care costs			
*Mental health care*	977 (2008)	2,863 (4957)	1877 (1,577 to 2,205)
*Other health care*	1,654 (1663)	1854 (2220)	201 (39 to 366)
*Medication*	324 (734)	220 (520)	−103 (−158 to −50)
*Subtotal costs*	2,954 (3058)	4,937 (5961)	1973 (1,562 to 2,350)
Productivity costs			
*Absenteeism*	3,549 (8886)	3,317 (6938)	−231 (−881 to 433)
*Presenteeism*	3,042 (4346)	2,163 (3429)	−876 (−1,208 to −556)
*Subtotal costs*	6,591 (10228)	5,481 (8321)	−1,107 (−1874 to −302)
Total societal costs	9,545 (10975)	10,417 (10436)	858 (−5 to 1753)

Health care costs and productivity costs were measured using the TIC-P instrument, and were converted to purchasing power parity corrected euros at the 2014 price level. Cost categories comprise all costs estimated for the period between baseline and 15 months follow-up. sd = standard deviation; 95% CI is the 95% confidence interval around the bootstrapped mean difference (5,000 samples). Total societal costs comprise all health care costs and productivity costs.

### 3.3 Effects

Table 3 presents the effect parameters of the economic evaluation in the two conditions. Each row in the table presents the data for each of five consecutive assessment waves. QALY results are cumulative over the current and all previous assessments. What can be observed from Table 3 is that the differential effect on Quality-adjusted life years (QALYs) between the two conditions (−0.016 – (−0.010) = 0.01) is not statistically significant (*p* = 0.69). The differential effect on depression-free days is statistically significant (*p* = 0.02) and indicates that the PCT group experienced more depression-free days then the CAU group.

**Table 3:** Cumulative quality of life gains over the course of the study.

Assessment	CAU m (sd)	PCT m (sd)
0 months	0	0
0–3 months	−0.006 (0.06)	0.005 (0.06)
3–6 months	−0.008 (0.08)	−0.005 (0.07)
6–12 months	−0.016 (0.11)	−0.013 (0.10)
12–15 months	−0.016 (0.10)	−0.010 (0.09)

m is mean; sd is standard deviation; Cum. QALY is the cumulative gain in QALYs since baseline.

### 3.4 Cost-utility and cost-effectiveness analysis

In Table 4, the costs, depression-free days and QALYs gained or lost over the 15-month follow-up period are presented for PCT and CAU. Also, the difference (increment) in costs between the two groups and the incremental cost-effectiveness ratio (ICER) with QALYs, respectively, depression-free days as the effect parameter is shown. The two plots positioned at the top of Figure 1 present two cost-effectiveness planes, with QALYs (top plane) and depression-free days (bottom plane) as the effect parameters. Each dot in the plane represents a bootstrapped mean ICER. By dividing the incremental costs by the incremental effects, the bootstrapped mean ICER of PCT compared with CAU from the societal perspective is calculated 15 months post-randomization.

**Table 4:** Results of the base case scenario cost-effectiveness analysis and alternative scenarios.

	CAU			PCT			Incremental cost-effectiveness ratio	
Analysis	Costs [m(95%CI)]	D-free days m	QALYs	Costs [m(95%CI)] euro	D-free days m	QALYs	D-free days (m)	QALYs (m)
Societal perspective, MI (base case)	9,506 (7599–11,759)	359	−0.015	10,354 (8611–12,515)	400	−0.010	20	141,451
Health care perspective, MI	2,948 (2375–3,558)	359	−0.015	4,867 (3969–6,180)	400	−0.010	47	378,922

All presented data are based on bootstrapped samples; ICER = incremental cost-effectiveness ratio; m is mean of the 5,000 bootstrapped samples; 95% CI is the 95% confidence interval of the 5,000 bootstrapped samples; D-free days is depression-free days.

Figure 1 shows that, after calculating the distribution of the ICERs over 4 quadrants, we found that there was a 47% likelihood that PCT led to additional QALYs at higher costs, and there was a 26% likelihood that PCT led to fewer QALY’s at higher costs. There was a 19% likelihood that PCT led to more QALYs at lower societal costs and an 8% likelihood that PCT led to less QALY’s at lower societal costs. When depression-free days was used as an outcome we found that PCT had a 72% probability of leading to more depression-free days at higher societal costs relative to CAU and a 27% probability of leading to more depression-free days at lower societal costs relative to CAU. There was a 1% likelihood that PCT led to less depression-free days at higher societal costs. At a WTP of ≥175.000 euro per QALY the probability of PCT being cost-effective compared to CAU was ≥50%. At WTP of ≥20 euro per depression-free day the probability of PCT being cost-effective was ≥50%.

### 3.5 Sensitivity analysis

To assess the sensitivity of our results to misspecifications of the key cost drivers, we repeated the analyses after raising and lowering the estimated total costs of each of the key cost categories by 20%. The sensitivity analysis was performed on the whole sample and based on the incremental cost-effectiveness ratio over 15 months. We found mental health care costs to be the most influential costs in these analyses and productivity costs were the second most influential.

## 4 Discussion

In this economic evaluation, the addition of Preventive Cognitive Therapy (PCT) to Care as Usual (CAU) was assessed in remitted patients who were treated with Cognitive Behavioral Therapy for their depression. With regard to costs, we found no differences between the PCT condition and the CAU condition. With regard to effects, we found no differences between the conditions in the number of gains in Quality-adjusted life years (QALYs) but a significant difference in the number of depression-free days. This is in line with our primary outcome where we found that adding PCT led to additional positive effects in delaying time to relapse/recurrence of depression over a period of 15 months among Cognitive Behavioral Therapy responders (19). Over the 15-month follow-up, the addition of PCT significantly delayed time to relapse/recurrence relative to CAU alone, HR = 1.807 (NNT = 8.1), *p* = 0.02 (95% CI = 1.029–3.174). The economic evaluation is slightly in favor of the PCT condition when depression-free days is used as an outcome. PCT is not cost-effective given the high costs per additional QALYs from the societal perspective when QALYs are the effect measure.

When QALYs gained was used as an outcome, we found that there was a 47% likelihood that PCT led to additional QALYs albeit at higher costs, and there was a 19% likelihood that PCT led to more QALYs at lower societal costs. At a Willingness To Pay of ≥175.000 euro per QALY the probability of PCT being cost-effective compared to CAU was ≥50%. In the Netherlands, a maximum “v-threshold” of 80,000 euro is used in decision-making. This threshold is only used for treatments targeted at diseases that cause a very high proportional loss of remaining health (40). The Willingness To Pay needed in this study far exceeds this threshold. An important consideration is that the 5-dimensional, 3-level EuroQol (EQ-5D) is not specific enough to detect differences in remitted patients with relatively few residual depressive symptoms. Furthermore, it only displays current health state and may not detect all the relapse/recurrences during the study. Moreover, the EQ-5D provides very limited coverage of themes identified by people with mental health problems (41). With that in mind, the main objective of PCT is to prevent relapse/recurrences by increasing the time to relapse/recurrence. Therefore, we also included the number of depression-free days as the outcome parameter in the cost-effectiveness analysis. We found that PCT had a likelihood of 27% of leading to more depression-free days at lower societal costs and a likelihood of 72% of leading to more depression-free days at higher societal costs than CAU. At Willingness To Pay of ≥20 euro per depression-free day the probability of PCT being cost-effective was ≥50%.

This study found no significant differences between the PCT and CAU conditions in costs. This is not surprising because these costs have a wide variation. However, PCT appeared to have slightly higher costs related to mental health care and CAU appeared to have slightly higher costs related to productivity losses. This is noteworthy since the PCT condition had the addition of eight sessions of PCT but these additional costs seem to be evened out by the medication and presenteeism costs in the CAU condition. A longer follow-up would be in favor of the PCT condition since the effects of the intervention are expected to last up to 5,5 to 10 years and the additional costs are once only (13). Furthermore, lowering the costs of providing PCT could be in favor of the PCT condition. This might be achieved by offering PCT in a group instead of individually, or by cutting therapists’ costs by training relatively less expensive therapists like nurse practitioners in primary care. Although, it is important to determine whether the effects of PCT remain the same if this is done. A previous study found that a supported self-help form of PCT was effective, but the effect size was smaller than PCT as offered by a specialist in Cognitive Behavioral Therapy. In addition, this supported self-help form of PCT was not cost-effective (15). In this study, the mean total cost was lower than in our study, but the differences between the treatment as usual and PCT groups were small.

This economic evaluation has several strengths as well as limitations. Strengths are that we reported the results in compliance with the consolidated health economic evaluation reporting standards (CHEERS) (22). Costs over the 15-month follow-up were assessed with questionnaires at 3, 6, 12 and 15 months, with the instruction to recall the last 2 weeks to minimize recall bias. Furthermore, drop-out rates were low (8.9%), as was the overall rate of missing data (6.5%) and compliance to the PCT intervention was high. Of the 97 patients who started the PCT, 98% received five or more sessions and 94% finished all eight sessions. A further strength of this study is that we specifically included Cognitive Behavioral Therapy responders, which allowed us to draw conclusions over a specific patient sample. However, it also leads to less generalizability in the general patient population. Although the effect of PCT has been demonstrated for other patient populations as well, that is patients that received diverse types of care, including Antidepression medication, care by a general practitioner and other types of psychosocial treatment (13–15, 42–44). A limitation of this study is that the 15-month follow-up might be too short to draw long-term conclusions about the cost-effectiveness of the PCT. Time to relapse/recurrence can exceed the 15-month follow-up. Since a recent study shows that the effect of PCT might last up to ten years, we expect a longer follow-up to be in favor of the PCT condition. More research with a longer follow-up is desirable to estimate the long-term cost-effectiveness. Furthermore, the data collected for this study are part of an RCT to examine the effectiveness of adding PCT to CAU. Therefore, the sample size was calculated to detect an effect in time to relapse/recurrences and may have been underpowered for an economic evaluation with cost-effectiveness / cost-utility analysis. We accounted for that by using probabilistic techniques. Finally, depression free days were measured with the SCID-I at the 15 month follow-up assessment therefore, recall bias is a possibility. Nevertheless, the SCID-I is a reliable measure (32) and research based on retrospective recall has shown that recall bias plays only a minor role (45).

In this economic evaluation from the societal perspective the addition of PCT to CAU in comparison to CAU alone was assessed in recovered patients who were treated with Cognitive Behavioral Therapy during their depression. We conclude that the economic evaluation is slightly in favor of the PCT condition when depression-free days is used as an outcome. PCT is not cost-effective given the high costs per additional QALYs from the societal perspective when QALYs are the effect measure. We recommend examining the long-term cost-effectiveness of PCT given the potentially sustained effects of PCT beyond our 15-month time horizon.

## Data availability statement

The raw data supporting the conclusions of this article will be made available by the authors, without undue reservation.

## Ethics statement

The studies involving humans were approved by Stichting Medisch-Ethische Toetsingscommissie Instellingen Geestelijke Gezondheidszorg (METiGG): NL34721.097.10. The studies were conducted in accordance with the local legislation and institutional requirements. The participants provided their written informed consent to participate in this study.

## Author contributions

MJ executed the study, organized the database and drafted this manuscript. MB performed the statistical analysis participated in writing the final manuscript. CB designed the study, wrote the protocol and participated in writing the final manuscript. MD provided substantial contributions to the acquisition of data and participated in writing the final manuscript. MK participated in writing the final manuscript. JD designed the study and participated in writing the final manuscript. All authors contributed to the article and approved the submitted version.
